# Microwave-Assisted Extraction of Anticancer Flavonoid, 2′,4′-Dihydroxy-6′-methoxy-3′,5′-dimethyl Chalcone (DMC), Rich Extract from *Syzygium nervosum* Fruits

**DOI:** 10.3390/molecules27041397

**Published:** 2022-02-18

**Authors:** Vachira Choommongkol, Khanittha Punturee, Piyatida Klumphu, Parintip Rattanaburi, Puttinan Meepowpan, Panawan Suttiarporn

**Affiliations:** 1 Department of Chemistry, Faculty of Science, Maejo University, Chiang Mai 50290, Thailand; vachira@mju.ac.th (V.C.); piyatida_kp@mju.ac.th (P.K.); 2 Cancer Research Unit of Associated Medical Sciences (AMS-CRU), Faculty of Associated Medical Sciences, Chiang Mai University, Chiang Mai 50200, Thailand; khanittha.taneyhill@cmu.ac.th; 3 Department of General Science, Faculty of Education, Nakhon Si Thammarat Rajabhat University, Nakhon Si Thammarat 80280, Thailand; parintip_rat@nstru.ac.th; 4 Department of Chemistry, Faculty of Science, and Graduate School, Chiang Mai University, Chiang Mai 50200, Thailand; pmeepowpan@gmail.com; 5 Center of Excellence in Materials Science and Technology, Faculty of Science, Chiang Mai University, Chiang Mai 50200, Thailand; 6 Faculty of Science, Energy and Environment, King Mongkut’s University of Technology North Bangkok, Rayong Campus, Rayong 21120, Thailand

**Keywords:** DMC, anticancer-rich extract, *Syzygium nervosum*, optimization, microwave-assisted extraction

## Abstract

2′,4′-Dihydroxy-6′-methoxy-3′,5′-dimethyl chalcone (DMC) is a biological flavonoid that is present in the fruits of *Syzygium nervosum* (Ma-Kiang in Thai). Microwave-assisted extraction (MAE), which utilizes microwave radiation to heat the extraction solvent quickly and effectively, was used to recover DMC-rich extract from *Syzygium nervosum* fruit. To determine the DMC content, a highly accurate and precise HPLC technique was developed. The influences of MAE conditions, including the solid–liquid ratio, microwave power, and microwave duration on the content of DMC, were sequentially employed by a single factor investigation and response surface methodology (RSM) exploratory design. The predicted quadratic models were fitted due to their highly significant (*p* < 0.0001) and excellent determination coefficient (R^2^ = 0.9944). The optimal conditions for producing DMC-rich extract were a ratio of sample to solvent of 1:35 g/mL, a microwave power of 350 W, and a microwave time of 38 min. Under the optimal MAE setting, the DMC content reached 1409 ± 24 µg/g dry sample, which was greater than that of the conventional heat reflux extraction (HRE) (1337 ± 37 µg/g dry sample) and maceration (1225 ± 81 µg/g dry sample). The DMC-rich extract obtained from MAE showed stronger anticancer activities against A549 (human lung cancer cells) and HepG2 (human liver cancer cells) than the individual DMC substance, which makes MAE an effective method for extracting essential phytochemicals from plants in the nature.

## 1. Introduction

*Syzygium* is a genus in the Myrtaceae family with over 1200 species worldwide. It is widely distributed in tropical Africa, Asia, Australia, New Caledonia, New Zealand, and the Pacific Islands, among other places [1]. The genus *Syzygium* is considered a source of bioactive phytochemical constituents, such as terpenoids [2], flavonoids [3], phenylpropanoids [4], chalcones [5], phenolic compounds [6], hydrolysable tannins, and chromone derivatives [7]. Furthermore, these bioactive compounds possess various pharmacological properties, including antifungal [8], antibacterial [9], antioxidant [10], anticancer [11], and antimicrobial activities [12].

**Figure 1:**
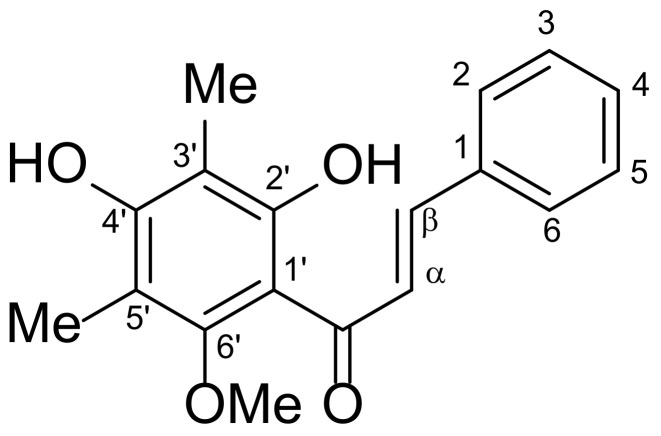
Structure of DMC.

*Syzygium nervosum* A. Cunn. ex DC., Ma-Kiang in Thai, is a local plant grown in the Northern region of Thailand. This plant is one of the important sources of medicinal herbs according to their chemical constituents. Flavonoids are one of the most common categories of natural health-promoting chemicals, and their diverse bioactivities play a significant role in human health [13]. In particular, 2′,4′-dihydroxy-6′-methoxy-3′,5′-dimethylchalcone (DMC) (Figure 1) is a biological active flavonoid isolated from *Syzygium campanulatum* Korth [11] and *Syzygium nervosum* [14]. DMC has been demonstrated to be responsible for anti-bacterial [15], anti-diarrheal [16], and anticancer activities [14].

Extraction is the first and seemingly most essential analytical procedure in the isolation of bioactive phytochemicals from medicinal plants prior to chromatographic detection [17]. Conventional procedures, such as maceration, refluxing, and Soxhlet extraction [18], have been applied for many decades for flavonoids extraction from various plants. However, these procedures are time-consuming and need substantial amounts of solvents. Other approaches, such as supercritical fluid extraction (SFE), ultrasonic-assisted extraction (UAE), and microwave-assisted extraction (MAE), have piqued attention as potential replacements for traditional methods [19]. Nowadays, MAE is commonly utilized to recover bioactive components from a variety of natural materials. MAE is a green extraction method, which is more beneficial and efficient than traditional extraction methods. Furthermore, this approach may be accomplished in less time, and with less organic solvent, wastewater removal, and energy use [20]. MAE was successfully used in the recovery of phenolic metabolites from Ma-Kiang [21] and flavonoid compounds from *Sedum aizoon* leaves [22].

The effort to enhance the potential of phytochemicals led to the formation of “rich extracts”, in which extracts possess bioactive substances. Several studies have found that biological-rich fractions have greater overall functional benefits in the management of chronic illnesses than single substances [23]. The rich bioactive fraction was obtained from the appropriate extraction conditions. Thus, the response surface method (RSM) was applied as a direction for optimization in chemical experiments or extraction operations. RSM based on the factorial designs of the central composite design (CCD) or Box–Behnken design (BBD) is a strategy for planning extraction experiments that assists researchers in developing models, analyzing the impacts of multiple factors, and achieving the optimum settings for maximum responses [24]. RSM has been demonstrated in multiple studies to be a beneficial strategy for optimizing the extraction of flavonoids from a variety of natural plants due to its superior design, which requires minimal experimentation to attain the expected results. For example, RSM was employed to improve the extraction performance of ultrasound-assisted flavonoid extraction from *Crinum asiaticum* by determining the optimal flavonoid extraction technique for increasing the flavonoid extraction rate [25]. Moreover, the effects of MAE conditions can be efficient extraction for flavonoids from *Apium graveolens* L. [26].

However, to the best of our knowledge, it was found that no reports were noticed in the literature on using MAE to extract the DMC anticancer compound from *Syzygium nervosum*. Based on our hypothesis, the quick MAE technique might be used to isolate bioactive-DMC extract from the plant, possibly enhancing the health benefit. Therefore, this is the first MAE study for DMC extraction from plant material that has focused on optimizing MAE parameters for the extraction of the DMC compound from *Syzygium nervosum* to obtain a DMC-rich fraction through RSM combined with BBD. Moreover, the simple and reproductive high performance liquid chromatography (HPLC) method for the determination of the DMC content was developed. Furthermore, a comparative extraction procedure was also performed to examine the differences in the DMC recovery from *Syzygium nervosum* among MAE, conventional HRE, and maceration. In addition, the anticancer activity of DMC-rich extract achieved under the optimal MAE conditions was compared to that of the DMC individual chemical in order to assess the potential for use as functional natural products for food components.

## 2. Results and Discussion

### 2.1. Method Validation of HPLC Determination

A regression analysis of the data displayed a superior linearity for the calibration curves of DMC over the concentration range of 0.25–100 µg/mL. The regression equation obtained from the experimental data was found to be y = 41481x − 9431.2. The excellent determination coefficient (R^2^) was observed to be 0.9999, indicating that the analyte concentration and peak area have a linear relationship. The values of limit of detection (LOD) and limit of quantification (LOQ) were 1.56 and 4.74 µg/mL, which indicate the sensitivity of the method. Precision was performed using the QC samples 0.25, 50, and 100 µg/mL because this range could be suitable for assessing DMC in *S. nervosum* fruits. The intra-day and inter-day precision for the determination of DMC using the HPLC method was also evaluated at three different concentration levels of the DMC standard by the DMC proposed procedure at different times of the same day and on five consecutive days (Table 1). The values of %RSD were found to be 0.67–5.75% for intra-day and 1.22–7.39% for inter-day precision. The low RSD values of the method precision were determined to be within the level of acceptance [27]. Accuracy is one of the most important parameters of analytical methodology. It can be determined by adding an exact amount of the DMC standard to *S. nervosum* extracts and expressing the result as a percent recovery. The recoveries shown in Table 1 were acceptable according to the AOAC (2002) recommendation (80–120%) [28]. The experimental results showed that the percentage of DMC recovery fulfilled this recommendation. The proposed method is highly accurate, as evidenced by the high percentage of DMC recovery.

### 2.2. Single Variables Experiment of DMC Microwave-Assisted Extraction

**Figure 2:**
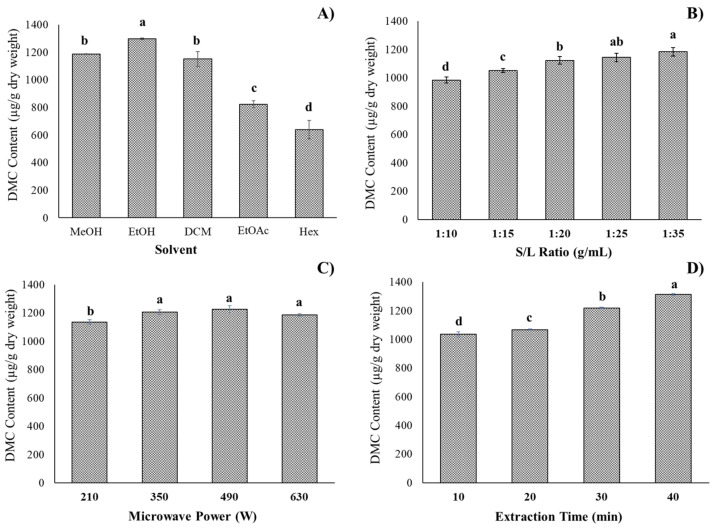
The effect of different types of solvent (**A**), solid-to-solvent ratio (**B**), microwave power (**C**), and microwave time (**D**) on DMC content from the fruits of *Syzygium nervosum* of MAE. Bar with the different letter show a significant difference (*p* < 0.05) among groups.

The selection of the solvent type is the most essential parameter in flavonoid extraction because the solvents used during the MAE operation are observed to have an impact on the value of flavonoid secondary metabolites extracted from plant material [29]. In this study, the impacts of five types of solvent (methanol, ethanol, dichloromethane, ethyl acetate, and hexane) on the DMC content were performed in the initial experiment. Figure 2A revealed that the flavonoid content of DMC varied depending on the type of solvent applied (640 ± 68 to 1298 ± 5 μg/g dry weight). In comparison to other solvents, ethanol produced the highest DMC yield (1298 ± 5 μg/g dry weight), which was verified to be statistically significant (*p* < 0.05). This is due to the polarity and viscosity of the solvent. The hydroxyl group of ethanol, which is hydrophilic, affects the solubility and intensity of the interaction with flavonoid in the samples [30]. Moreover, ethanol has a remarkable ability to absorb microwave energy [31]. Thus, microwave radiation is exploited to heat ethanol, which interfaces with plant materials, resulting in the partition of the target flavonoid from the fruits of *S. nervosum* into the solvent. As a result, ethanol was employed as a solvent in the MAE extraction process of DMC, not only because of its extraction effectiveness but also because it is safe for human edibles and is environmentally friendly.

To investigate the influence of various plant-to-solvent ratios on the DMC amount, the ratios were set at 1:10, 1:15, 1:20, 1:25, and 1:35 g/mL, while the other variables of the experiment remained constant (Figure 2B). As the solid-to-liquid ratio changed from 1:10 to 1:20 g/mL, the DMC content was observed from 985 ± 21 to 1123 ± 27 μg/g dry weight. After that, the DMC yield slightly increased, with an increase in ratio to 1:35 g/mL (1184 ± 30 μg/g dry weight). It should be noted that when the solid-to-liquid ratio increases, the DMC content increases as well, reaching 1:35 g/mL. The explanation for this behavior is that when the solvent volume increases, the encounter surface area between the fruit of *S. nervosum* and the ethanol solvent expands, as it can dissolve and allow for substantial extraction to occur [32]. For extensive evaluation, the range of solid-to-liquid ratios was chosen to be from 1:15 to 1:35 g/mL for further RSM optimization.

The influence of microwave power on the DMC content was examined by varying the microwave power (210, 350, 490 and 630 W), while the other experimental parameters were held constant. As shown in Figure 2C, the content of DMC varied from 1136 ± 15 to 1227 ± 24 µg/g dry weight. It could be observed that the DMC content increased when the microwave power increased from 210 W to 490 W and remained stable with an increase in microwave power to 630 W. This is due to the increased microwave power, which has the potential to improve the extraction efficiency by strengthening the interfacial interactions between the electromagnetic field and the DMC analyte of the fruits of *S. nervosum*.

The effects of various microwave extraction times (10, 20, 30, and 40 min) on the DMC content were investigated while other factors were fixed. Figure 2D illustrates that the DMC content increased from 1034 ± 18 to 1313 ± 7 µg/g dry weight when the extraction period was raised from 10 to 40 min. During the DMC extraction process, MAE may readily rupture the plant’s cell wall and release the highest quantity of flavonoid components in the shortest period of time [33]. However, the long extraction times may cause flavonoids to decompose due to more energy consumption [34]. As a result, the extraction time range of 10–40 min was chosen for future investigations.

### 2.3. Fitting the Model

The influences of MAE parameters (material-to-ethanol ratio, energy power, and microwave time) on the amount of flavonoid, DMC, were investigated using the Box–Behnken design (BBD). The RSM experimental design and DMC contents of each condition are illustrated in Table 2. The DMC values varied from 1145 to 1365 µg/g dry sample.

ANOVA was used to examine the DMC model’s adequacy and fitness (Table 3). Based on the ANOVA result, the DMC model is notable based on the F-value of 98.01. An F-value of this level has a 0.01% probability of appearing due to noise. Significant model terms have a *p*-value of less than 0.0500. Thus, the DMC model was remarkably significant (*p* < 0.0001). The lack of fit F-value of 1.83 indicates that lack of fit is not significantly related to the pure error.

A non-significant lack of fit of the DMC model is acceptable. Moreover, the linear effect of the solid-to-liquid ratio (X_1_), microwave power (X_2_), and microwave time (X_3_), as well as the quadratic (X_2_^2^ and X_3_^2^), have a remarkably significant influence on the extraction of the DMC content (*p* < 0.05).

**Figure 3:**
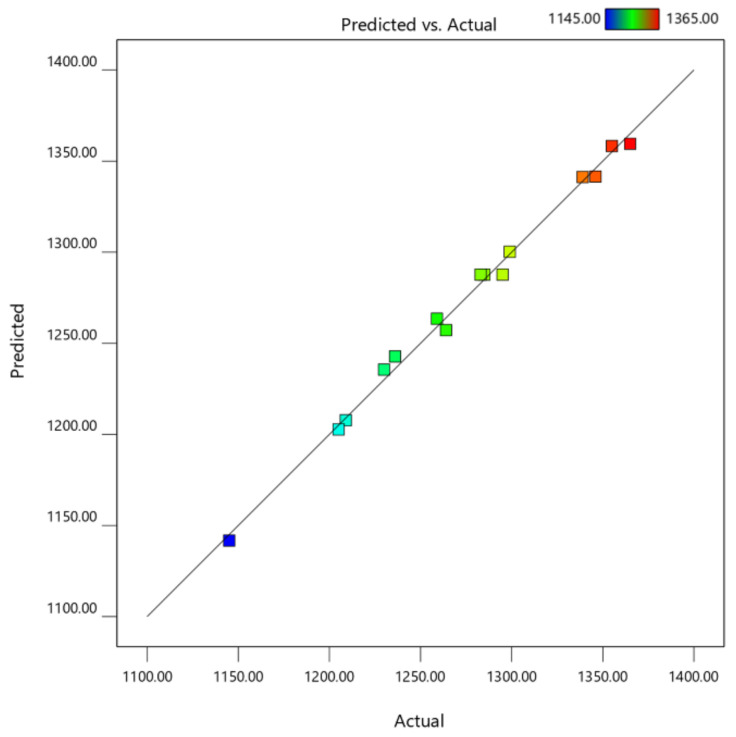
Plots comparing the experiment’s actual values versus predicted values.

The values of R^2^, Adj-R^2^, and Pre-R^2^ (0.9944, 0.9842, and 0.9305, respectively) were all close to 1. The adjusted R^2^ of 0.9842 is reasonably close to the predicted R^2^ of 0.9305. The difference is less than 0.2. This indicated excellent correlation among both predicted and actual results, and the DMC extraction prediction was well-fit by the polynomial model [22]. Moreover, Figure 3 demonstrates that the measured values matching the predicted and actual values were dispersed around the 45-degree line in close enough proximity. It is suggested that the actual results may be correctly predicted from model parameters that contribute to the quadratic model’s relative accuracy. In addition, the minimal values of the coefficient of variation (CV = 0.6171%) indicated that the experimental value was consistent and repeatable [35]. Thus, the model could be used to explore the design parameters and to predict the content of the DMC in the extracts. The second-order polynomial equation (Equation (1)) of the DMC content was expressed as follows:

Y = 1287.67 + 50.50X_1_ − 11.50X_2_ + 57.75X_3_ + 2.50X_1_X_2_ − 9.00X_2_X_3_ − 0.33X_1_^2^ + 12.67X_2_^2^ − 37.33X_3_^2^ (1)

Y is replaced by a DMC content (µg/g dry sample);
X_1_ is replaced by a solid-to-liquid ratio (g/mL);
X_2_ is replaced by a microwave power (W);
X_3_ is replaced by a microwave time (min).

When the independent variables are in the experimental ranges; X_1_ in 1:15 to 1:35, X_2_ in 350 to 630 W, and X_3_ in 10 to 40 min, the equality is suitable for predicting the DMC production.

### 2.4. Analysis of Response Surface and Optimization of MAE Process by RSM

**Figure 4:**
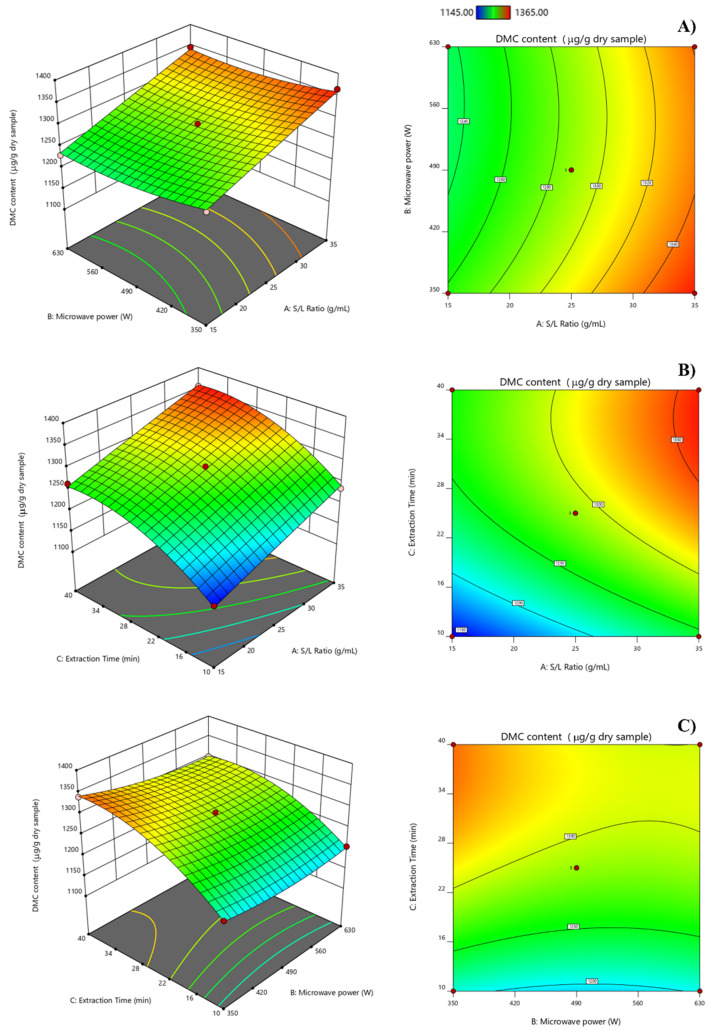
Response surface (3D) and contour (2D) plots describe the interactive impacts of MAE parameters; (**A**) solid-to-liquid ratio (X_1_) and microwave power (X_2_); (**B**) solid-to-liquid ratio (X_1_) and microwave time (X_3_); and (**C**) microwave power (X_2_) and microwave time (X_3_).

As shown in Figure 4, the quadratic polynomial equation of the DMC content can be visually displayed by DMC response surface (3D) and contour (2D) plots. The graphs were explored by altering two parameters across a DMC response while leaving the remaining factor to center level constant. The effects of the sample-to-solvent ratio (X_1_), energy power (X_2_), and their mutual interaction on the DMC content were observed first, as shown in Figure 4A. The maximum DMC yield was achieved at the ratio of Ma-Kiang-powder-to-ethanol of 1:30–1:35 g/mL and at a microwave power of 350–420 W. A higher volume of ethanol may enter the cells of Ma-Kiang fruits as the solid-to-liquid ratio rises, causing the cell wall of fruits to rupture and allowing for more bioactive chemicals to be recovered [36]. The influences of the plant-to-solvent ratio (X_1_) and microwave time (X_3_) on the response are shown in Figure 4B. Increasing the Ma-Kiang-powder-to-ethanol ratio from 1:30 to 1:35 g/mL with the microwave time ranging from 35 to 40 min resulted in an enhancement of the DMC. As illustrated in Figure 4C, a 3D response surface plot was constructed for DMC extraction with a varying microwave power (X_2_) and microwave extraction duration (X_3_). It can be shown that when the microwave radiation energy and extraction duration are at their maximum values of 350–420 W and 35–40 min, respectively, maximal DMC recovery can be accomplished. When a longer microwave duration was expected for solvent penetration into plant tissue, increasing the microwave power might be ascribed to a higher solubility of bioactive chemicals. As a result of the increased microwave radiation and treatment time, the temperature inside the enclosed microwave vessel can be kept at high levels, facilitating cell rupture and increasing the bioactive component release into the extraction solvent [37].

The RSM ability employed Design Expert V12 Trial Software to identify the optimum settings of the MAE independent parameters with the highest DMC response. The results suggested that the highest recovery of DMC may be reached when the material-to-solvent ratio, microwave radiation energy, and microwave time are 1:35 g/mL, 350 W, and 38 min. The maximal expected yield of DMC under these settings was 1389 µg/g dry sample.

### 2.5. Verification of the DMC Predictive Model and Comparison of MAE with Conventional Methods

Following that, to evaluate the accuracy and acceptability of the extraction process, a verification of the MAE process was carried out. The software’s anticipated parameters were to be a solid-to-liquid ratio of 1:35 g/mL, energy power of 350 W, and microwave time of 38 min. The actual DMC content in experiments was determined to be 1409 ± 24 µg/g dry sample after a series of triple trials under the obtained conditions, which was close to the model’s expected value (1389 µg/g dry sample) with a 1.44% error (Table 4). It is indicated that the quadratic model accurately predicted the empirical results of the MAE procedure.

Various techniques are employed to extract various natural compounds, the amount of bioactive components, and the extraction efficiency. In comparison to the different extraction methods, MAE produced the highest DMC yield (1409 ± 24 µg/g dry sample), followed by HRE (1337 ± 37 µg/g dry sample), and maceration (1225 ± 81 µg/g dry sample), as shown in Table 4. Furthermore, the MAE requires only 38 min to extract the DMC, which takes much less time than HRE (2 h) and maceration (20 h). According to previous research, MAE produced more flavonoid from *Radix astragali* than UAE and HRE [38]. The results showed that MAE had a shorter microwave time and increased DMC yields than the other approaches. Due to clean energy transfer to the matrix, microwave radiation might speed up the extraction process and the extraction efficiency might increase [39]. Furthermore, the direct absorption of microwave radiation by the material resulted in an energy-saving. When compared to the HRE process, there is energy waste and less selectivity due to heat being transferred from the heating source (outside) to the inside of the sample [39]. In addition, the MAE technique performed better in comparison to maceration for the recovery of phenolic and flavonoid compounds from *Cassia alata* [40] and for the extraction of phenolic compounds from *Melastoma sanguineum* fruit [41]. Although HRE and maceration are the solid–liquid conventional processes for the extraction of flavonoids that are still used today, MAE might be a viable option for extracting the DMC-rich extract from the fruits of *S. nervosum.*

### 2.6. Cell Viability

**Figure 5:**
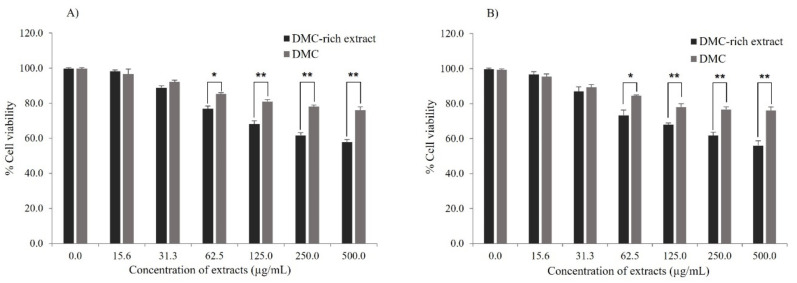
The effects of DMC-rich extract and DMC of *S. nervosum* on A549 cell viability at (**A**) 24 h and (**B**) 48 h. The data are an average of %cell viability ± SD of three studies. * *p* < 0.05 and ** *p* < 0.01 define statistically significant different.

**Figure 6:**
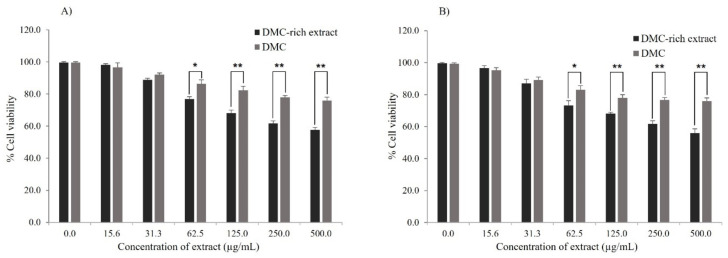
The effects of DMC-rich extract and DMC of *S. nervosum* on HepG2 cell viability at (**A**) 24 h and (**B**) 48 h. The data are an average of %cell viability ± SD of three studies. * *p* < 0.05 and ** *p* < 0.01 define statistically significant different.

The anti-cancer activity of DMC-rich extract obtained by MAE process and isolated DMC obtained from a chromatographic technique of *S. nervosum* fruits was investigated in A549 (human lung cancer cells) and HepG2 (human liver cancer cells) by using a MTT assay. The results showed a similar trend in both A549 and HepG2 cells. The viability of A549 and HepG2 cells was significantly reduced by DMC-rich extract and individual DMC in all treatments. However, the DMC-rich extract of *S. nervosum* showed stronger anti-cancer activity than individual DMC at a concentration of extract equal to or more than 62.5 µg/mL in both 24 and 48 h of incubation (Figure 5 and Figure 6). In order to test the specificity of DMC and DMC-rich extract against cancer cells, normal human PBMCs were also tested. Both DMC and DMC-rich extract at concentrations of up to 500 µg/mL had no cytotoxicity to normal human PBMCs, as the percentage of cell viability was close to 100% (data not shown).

According to a previous study, DMC showed a recordable cytotoxicity to P-388, KB, HT29, MCF-7, A549, ASK, Hek293, HepG2, Hela, Hela/Tax, and Vero cell lines [14,42]. In this study, the IC_50_ of DMC against A549 and HepG2 cells was more than 500 µg/mL, which was different from the previous study (IC_50_ of DMC equal to 13.04 µg/mL (A549) and 2.48 µg/mL (HepG2)) [14,42]. This might be caused by various factors, such as the difference in cell culture condition, the method of cytotoxicity testing, and the amount of cells.

The DMC-rich extract obtained from MAE may promote the anticancer potential of chemotherapies through combination strategies. The use of chemical combinations can be therapeutic to the biological systems that they are designed towards, as in the case of combination therapy [43]. A whole plant or mixes of plants are employed in traditional medicine rather than individual components. At an equivalent dosage, there is a suggestion that crude natural plant extracts have a higher efficacy in in vitro or in vivo activity than separated components [44]. Thus, the rapid MAE process has the potential to improve the activity of existing pharmaceutical preparations, as well as the efficacy of natural product therapies.

## 3. Materials and Methods

### 3.1. Materials and Apparatuses

The fresh fruits of *S. nervosum* were collected from Doi Saket province, Chiang Mai, Thailand, during August–September 2019. The *S. nervosum* was identified from the Forest Herbarium, Department of National Parks, Wildlife and Plant Conservation, Ministry of Natural Resources and Environment, Bangkok, Thailand (Specimen voucher No. 147924). The fruits were left to dry in an oven at 55–60 °C until they reached a consistent weight. The moisture content of the dried plant material was 13.14% ± 0.35 using AOAC method of No. 927.05, (AOAC, 2000). Subsequently, the dry fruits were ground with an electrical blender (E3TB1-200K, Electrolux, Bangkok, Thailand) and passed through a 45-mesh sieve and stored in room temperature.

Commercial grade solvents, including methanol, 95% ethanol, ethyl acetate, dichloromethane, and hexane, were purified before being used as extraction and purification solvents. RCI labscan (Bangkok, Thailand) provided the deionized water, HPLC grade methanol, and phosphoric acid. A purification procedure was carried out utilizing PTLC plates with silica gel (silica gel 60 PF_254_, Merck, Darmstadt, Germany). The melting point was performed by melting point analyzer (Gallenkamp, Manchester, UK). The ^1^H- and ^13^C-NMR operations were investigated by NMR spectrometers (DRX 400 MHz, Bruker, Billerica, MA, USA) in CDCl_3_.

Quantitative analysis was performed by a Perkin Elmer Flexar^tm^ HPLC system (PerkinElmer, Waltham, MA, USA) coupled with photodiode array detector (PAD) (PerkinElmer, Waltham, MA, USA). The PerkinElmer Chromera^@^ CDS software operated the instruments and analyzed/processed the data.

### 3.2. Isolation and Characterization of Standard DMC Compound

Extraction and isolation of DMC from the fresh fruits of *S. nervosum* were carried out as modified method of previously study by Chailungka et al., 2017 [14]. The fruit powder (30 g) was macerated with 95% ethanol (500 mL) at room temperature for 20 h. The extract solution was filtered and evaporated until it was completely dry. To prepare dichloromethane crude extract, the dried crude product was dissolved in dichloromethane. To extract DMC enhanced fraction, the crude product was subjected to purification using preparative thin-layer chromatography (PTLC; silica gel) with acetone/hexane = 1:9 as eluent. The fraction was then purified again by PTLC to obtain a high yield of 2′,4′-dihydroxy-6′-methoxy-3′,5′-dimethylchalcone (DMC) (0.0378 g, 0.12% yield).

The main characterization of DMC is shown as follows: yellow solid, melting point 120.5–122.3 °C, ^1^H-NMR (400 MHz, CDCl_3_, δ, ppm) whose chemical shifts are expressed as δ-values in parts per million (ppm) unit with downfield from tetramethyl silane (TMS: δ 0.00) and relative to residue CHCl_3_ as the internal reference (^1^H: δ 7.26, ^13^C: δ 77.00): 2.13 (s, 3H, CH_3_-5′), 2.16 (s, 3H, CH_3_-3′), 3.66 (s, 3H, OCH_3_-6′), 5.48 (s, 1H, OH-4′), 7.41 (m, 3H, Ph-H-3,4,5), 7.65 (m, 2H, Ph-H-2,6), 7.84 (d, *J* = 15.7 Hz, 1H, H-b), 7.99 (d, *J* = 15.7 Hz, 1H, H-a), 13.62 (s, 1H, OH-2′), ^13^C-NMR (100 MHz, CDCl_3_, δ, ppm): 7.6, 8.2, 62.4, 106.5, 108.9, 109.0, 126.7, 128.4(2C), 128.9(2C), 130.2, 135.3, 142.9, 158.8, 159.6, 162.0, 193.4 (Figure S1). The spectroscopic data of DMC were in agreement with previously reported data [14]. Based on the ^1^H-, ^13^C-NMR spectrum and HPLC chromatogram, the obtained DMC (isolated) had a >99% purity. It was further used as the standard compound for validation of the analytical method and bioactivity investigation.

### 3.3. HPLC Conditions and Method Validation for Quantification of DMC

Determination of DMC in *S. nervosum* fruit extracts was analyzed by a Perkin Elmer Flexar^tm^ HPLC system (Perkinelmer, Waltham, MA, USA) equipped with a quaternary pump, a photo diode array detector (Perkinelmer, Waltham, MA, USA), and a column oven. A reversed-phase LC separation was performed by C18 Brownlee column (4.6 mm, 250 mm, 5 µm, PerkinElmer, Waltham, MA, USA) maintained at 35 °C with an isocratic elution. The detector wavelength was set at 340 nm. The injection volume was set at 10 µL. A mixture of methanol and 0.1% phosphoric acid in water (80:20, *v/v*) was used as the eluent at a flow rate of 1.0 mL/min. Peaks appearing in chromatogram were identified by comparing retention durations to those of the DMC standard chemical (Figure S2).

The linearity LOD, LOQ, precision, and accuracy of the HPLC analysis were all validated. Linearity was assessed by preparation of serially diluting the DMC stock solutions to yield eight concentration levels of DMC solution (0.25, 0.5, 1, 5, 10, 25, 50, and 100 µg/mL, five replicates each). The DMC calibration curve was established by constructing the graph between the peak area of DMC (y-axis) and the concentrations of DMC (x-axis). Regression analysis was performed to fit the linear curve and to obtain the calibration equation and correlation coefficient. The standard deviation (S_a_) of the intercept, with y-axis of peak area and the slope (b) of the DMC calibration curve, was used to calculate the limit of detection (LOD = 3.3 × S_a_/b) and limit of quantification (LOQ = 10 × S_a_/b).

The precision method was assessed using QC sample of DMC standard (0.25, 50, and 100 µg/mL). Assay of five samples of each concentration was used for assay intra-day precision. Daily assays of the samples for five days were examined for inter-day precision. The relative standard deviation (RSD) values of DMC content in five replicates were determined. The accuracy of method was investigated by spiking standards of DMC in extract solution at three different concentration levels (0.25, 50, and 100 (µg/mL) and calculating the percent recovery [% Recovery = (C_1_−C_2_)/C_3_ × 100, where C_1_ is concentration of DMC spiked extract, C_2_ is concentration of unspiked extract, and C_3_ is concentration of spiked DMC standard].

### 3.4. Microwave-Assisted Extraction (MAE) Approach of DMC-Rich Extract

The MAE process was investigated using an Electrolux EME2024MW system (Electro-lux, Bangkok, Thailand) with a reflux apparatus. The *S. nervosum* fruit powder and solvent were prepared in a 500 mL glass extraction vessel. The values of all parameters, including extraction solvents, sample-to-solvent ratio, microwave energy, and microwave irradiation time, were set according to the single factor experiment and RSM design. The vessel was inserted inside the microwave cavity and fitted with a condenser. After extraction in the microwave oven, the mixture was filtered after being cooled at room temperature. The supernatant solution was carefully collected and then diluted 5-fold with the same solvent used for extraction. Prior to high-performance liquid chromatography (HPLC) measurement of DMC, the solution was filtered through a 0.45 μm pore nylon syringe filter.

In order to optimize the MAE conditions of DMC from *S. nervosum* fruits, the MAE parameters that influenced the most the DMC recovery were sequentially examined by single variable experiments and RSM exploratory design to obtain a DMC-rich extract. Moreover, the results of the optimal MAE process were compared to traditional HRE and maceration methods.

### 3.5. Single Variable Investigation

Effect of four independent MAE process parameters, including solvent types, sample–solvent ratio, microwave power, and extraction time on extraction efficiency, were investigated. Methanol (MeOH), 95% ethanol (EtOH), dichloromethane (DCM), ethyl acetate (EtOAc), and hexane (Hex) were utilized as extraction solvents to evaluate solvent efficiency in the first phase based on operational performance. In addition, the ratio of *S. nervosum* fruits to ethanol (1:10, 1:15, 1:20, 1:25, and 1:35 g/mL), microwave energy (210, 350, 490 and 630 W), and MAE duration (10, 20, 30, and 40 min) were also investigated. The DMC content was represented as mean of DMC content ± standard deviation (SD) for three replicates. The studies determined a suitable set of independent variables for RSM experiment design.

### 3.6. Experimental Design

The response surface methodology (RSM) was used to construct the computed functional correlation between a DMC response variable and MAE variables, as well as to select the optimal MAE factors for a maximum response. The RSM’s Box–Behnken design (BBD) was used to design the minimum number of experiments to achieve the appropriate parameters for the MAE process. The suitable values of the coded independent variables, the ratio of solid–liquid (X_1_), microwave power (X_2_), and MAE duration (X_3_), were set based on the experimental data of the single-factor operations, as indicated in Table 5. On the basis of BBD, a total of 15 experiments were conducted using various combinations of three MAE parameters by Design Expert V12 Trial Software (Stat-Ease Inc., Minneapolis, MN, USA). In addition, the content of DMC (Y) was chosen as the response, and its values were averaged among the triplicates in each experiment.

The statistical significance of the model was assessed using ANOVA and response surface analysis. To anticipate the optimal MAE parameters of DMC, a second-order polynomial model concerning the correlation between the MAE independent and DMC response variables (Equation (2)) was constructed using the determination coefficient (R^2^), adjusted determination coefficient (R^2^ adj), and coefficient of variance (CV%).

(2) $$Y=\beta_{0}+\sum_{i=1}^{3}\beta_{i}X_{i}+\sum_{i=1}^{3}\beta_{\mathrm{ii}}X_{i}^{2}+\sum_{i=1}^{2}\sum_{j=i+1}^{3}\beta_{\mathrm{ij}}X_{i}X_{j}+\varepsilon$$

where Y is the DMC response; X_i_, X_i_^2^, and X_i_X_j_ are the linear, quadratic, and interaction terms of the coded MAE independent variables, respectively; β_0_ is the equation parameter for the constant term; β_i_, β_ii_, and β_ij_ are the coefficients for the intercept, linear, quadratic, and interaction terms, respectively; and ε is a random error.

### 3.7. Verification of the DMC Predictive Model

The model validity of the MAE optimized experimental value was confirmed by repeating the extraction three times using the optimized MAE setting. The actual DMC yield was proven by comparing the anticipated DMC value with the experimental DMC content. The percentage of error was calculated [percentage error = (measured value − predicted value)/predicted value × 100].

### 3.8. Conventional Maceration and Heat Reflux Extraction (HRE)

Maceration was performed in a 500 mL Erlenmeyer flask covered with aluminum foil to minimize evaporation and degradation. Ethanol was chosen as the solvent, the ratio of *S. nervosum* fruits to ethanol was fixed at 1:35 (g/mL), and samples were taken at 20 h at room temperature (25 °C). HRE was also conducted under reflux, the solid-to-liquid ratio was 1:35 g/mL, and the temperature was fixed during the extraction at the ethanol boiling point for 2 h [38]. The extract solutions were then filtered through a 0.45 µm pore nylon syringe filter and carefully collected for further DMC analysis by HPLC.

### 3.9. Statistical Analysis

Each DMC extraction conditions was performed three times, and the measured DMC contents were represented as their mean ± SD. Statistical variations among DMC extraction conditions were estimated using one-way analysis of variance (ANOVA), and significant differences were determined using post hoc testing (least significant difference, or LSD, defined at *p* < 0.05) with SPSS software, version 17.0 (SPSS Inc., Chicago, IL, USA).

### 3.10. Cell Lines and MTT Assay

A549 (human lung cancer cells) and HepG2 (human liver cancer cells) cells were generously supported by Dr. Ratchada Cressey and Dr. Preeyanat Vongchan (Chiang Mai University, Chiang Mai, Thailand; cell lines were bought from American Type Culture Collection (ATCC)), respectively. A549 and HepG2 cells were grown in Dulbecco’s modified Eagle medium (DMEM) containing 10% of fetal bovine serum (FBS) and antibiotics (100 µg/mL of streptomycin and 100 U/mL of penicillin) until reaching 80% confluence. This stage of cells was used for cytotoxicity testing by MTT assay. Besides cancer cells, human peripheral blood mononuclear cells (PBMCs) were also used as normal control cells. PBMCs were prepared from 3 healthy donors by Ficoll-Hypaque gradient centrifugation and cultured in RPMI medium containing 10% of FBS and antibiotics (100 µg/mL of streptomycin and 100 U/mL of penicillin).

The 3-(4,5-dimethylthiazol-2-yl)-2,5-diphenyltetrazolium bromide (MTT) assay was applied to investigate the cytotoxic effect of DMC-rich extract and individual DMC on cancer (A549 and HepG2) and normal (human PBMCs) cells. Cancer (1 × 10^4^ cells/well) or human PBMCs (1 × 10^5^ cells/well) were introduced to a 96-well tissue culture plate. DMC-rich extract and individual DMC were dissolved in dimethyl sulfoxide (DMSO) and diluted to desire concentrations. Different concentrations of extracts were added into each well. Cells were then incubated at 37 °C in a 5% CO_2_ incubator for 24 or 48 h. After incubation, 20 µL of MTT solution (5 mg/mL of MTT in PBS, pH 7.4) was added into each well. The plate was then further incubated at 37 °C for another 4 h. Culture medium was drained out and the formazan dye crystal was dissolved with 100 µL of DMSO. Absorbance was determined at 540 nm by using microplate reader. Three independent measurements were used to determine each result. The cell viability data were represented as a percentage when compared to the control group (100%).

## 4. Conclusions

Microwave-assisted extraction (MAE) is successfully applied for the separation of DMC-rich extract from *Syzygium nervosum* fruits using ethanol as the solvent. The highest extraction efficiency was obtained (1409 ± 24 µg/g dry sample) when the sample-to-solvent ratio, microwave power, and microwave time were 1:35 g/mL, 350 W, and 38 min, respectively. The acquired extraction DMC content, according to the current MAE approach, was greater than HRE (1337 ± 37 µg/g dry sample) and maceration (1225 ± 81 µg/g dry sample). The DMC-rich extract obtained from MAE shows stronger anti-cancer activity than the individual DMC compound; hence, the combined actions of the phytochemicals may be partly responsible for the activities. Both compounds were not toxic to normal human PBMCs, indicating that they were specific to A549 and HepG2 cells. Due to its reduction in the process time and solvent waste, the MAE approach is considered as an efficient alternative procedure for the extraction of DMC when compared to its traditional maceration method.

## Figures and Tables

**Table 1:** Analytical of intra-day and inter-day precision assay and analytical recovery of DMC standard solution were added to samples.

Concentration of DMC (µg/mL)	%RSD		%Recovery (µg/mL ± SD)
	Intra-Day	Inter-Day	
0.25	5.75	7.39	85.3 ± 1.5
50	1.36	1.87	98.5 ± 2.5
100	0.67	1.22	97.3 ± 3.8

**Table 2:** Box–Behnken design (BBD) for optimizing MAE process from the fruits of *S. nervosum* for each variable with their observed responses.

Standard Order	Extraction Variables			DMC Content (µg/g Dry Sample)
	X_1_: Solid-to-Liquid Ratio (g/mL)	X_2_: Microwave Power (W)	X_3_: Microwave Time (min)	
2	1:35	350	25	1365
10	1:25	630	10	1205
5	1:15	490	10	1145
7	1:15	490	40	1264
13	1:25	490	25	1295
8	1:35	490	40	1355
4	1:35	630	25	1346
9	1:25	350	10	1209
11	1:25	350	40	1339
15	1:25	490	25	1283
1	1:15	350	25	1259
14	1:25	490	25	1285
6	1:35	490	10	1236
3	1:15	630	25	1230
12	1:25	630	40	1299

**Table 3:** ANOVA of the DMC content’s anticipated regression analysis.

Source	Sum of Squares	Degree of Freedom	Mean Square	F-Value	*p*-Value	Remark
Model	54,540.17	9	6060.02	98.01	<0.0001	significant
X_1_-S-to-L Ratio	20,402.00	1	20,402.00	329.95	<0.0001	significant
X_2_-Microwave Power	1058.00	1	1058.00	17.11	0.0090	significant
X_3_-Microwave Time	26,680.50	1	26,680.50	431.49	<0.0001	significant
X_1_X_2_	25.00	1	25.00	0.4043	0.5528	
X_1_X_3_	0.00	1	0.00	0.0000	1.0000	
X_2_X_3_	324.00	1	324.00	5.24	0.0707	
X_1_²	0.4103	1	0.4103	0.0066	0.9382	
X_2_²	592.41	1	592.41	9.58	0.0270	significant
X_3_²	5146.26	1	5146.26	85.23	0.0003	significant
Residual	309.17	5	61.83			
Lack of Fit	226.50	3	75.50	1.83	0.3729	not significant
Pure Error	82.67	2	41.33			
Cor Total	54,849.33	14				
R^2^	0.9944					
R^2^ (adj)	0.9842					
R^2^(pred)	0.9305					
CV (%)	0.6171					
Standard Deviation	7.86					

**Table 4:** Predicted value, experiment confirmation of the optimal MAE condition, and comparison of MAE with conventional extraction methods.

Extraction Method	Optimal MAE Conditions			Maximum Value (µg/g Dry Sample)	
	X_1_	X_2_	X_3_	Predicted Value	Experimental Value
MAE	1:35 g/mL	350 W	38 min	1389	1409 ± 24 ^a^
HRE	1:35 g/mL	-	2 h	-	1337 ± 37 ^a^
Maceration	1:35 g/mL	-	20 h	-	1225 ± 81 ^b^

The data are provided as mean value ± standard deviation (*n* = 3). Values preceded by different letters in the same column are markedly different (*p* < 0.05).

**Table 5:** The uncoded MAE independent variables and levels for Box–Behnken design.

Independent Variable	Variable Symbol	Level		
		−1	0	1
Sample–Solvent ratio (g/mL)	X_1_	1:15	1:25	1:35
Microwave Power (W)	X_2_	350	490	630
Microwave Time (min)	X_3_	10	25	40

## Data Availability

Data sharing not applicable.

## Supplementary Materials

The following supporting data can be downloaded online, Figure S1: (A) ^1^H-NMR and (B) ^13^C-NMR spectrum of DMC; Figure S2: HPLC Chromatogram of (A) DMC-rich extract and (B) standard of DMC.
