# Supplementary material for: Microwave-Assisted Extraction of Anticancer Flavonoid, 2′,4′-Dihydroxy-6′-methoxy-3′,5′-dimethyl Chalcone (DMC), Rich Extract from Syzygium nervosum Fruits
Source: Molecules. 2022 Feb 18;27(4):1397. doi: 10.3390/molecules27041397 (PMC8877704; doi:10.3390/molecules27041397)
Supplement: Supplementary file 1 [file molecules-27-01397-s001.zip › molecules-1560135-supplementary.pdf]

Supplementary Material

Microwave-Assisted Extraction of Anticancer Flavonoid, 2',4'-Dihydroxy-6'-Methoxy-3',5'-Dimethyl Chalcone (DMC), Rich Extract from *Syzygium Nervosum* Fruits

Vachira Choommongkol [1], Khanittha Punturee [2], Piyatida Klumphu [1], Parintip Rattanaburi [3], Puttinan Meepowpan [4,5] and Panawan Suttiarporn [6,*]

1. Department of Chemistry Faculty of Science, Maejo University, Chiang Mai 50290, Thailand; vachira@mju.ac.th and piyatida_kp@mju.ac.th
2. Cancer Research Unit of Associated Medical Sciences (AMS-CRU), Faculty of Associated Medical Sciences, Chiang Mai University, Chiang Mai 50200, Thailand; khanittha.taneyhill@cmu.ac.th
3. Department of General Science, Faculty of Education, Nakhon Si Thammarat Rajabhat University, Nakhon Si Thammarat 80280, Thailand; parintip_rat@nstru.ac.th
4. Department of Chemistry, Faculty of Science, and Graduate School, Chiang Mai University, Chiang Mai 50200, Thailand p.meepowpan@cmu.ac.th
5. Center of Excellence in Materials Science and Technology, Faculty of Science, Chiang Mai University, Chiang Mai 50200, Thailand; p.meepowpan@cmu.ac.th
6. Faculty of Science, Energy and Environment, King Mongkut's University of Technology North Bangkok, Rayong campus 21120, Thailand; panawan.s@sciee.kmutnb.ac.th*

*Corresponding author: panawan.s@sciee.kmutnb.ac.th

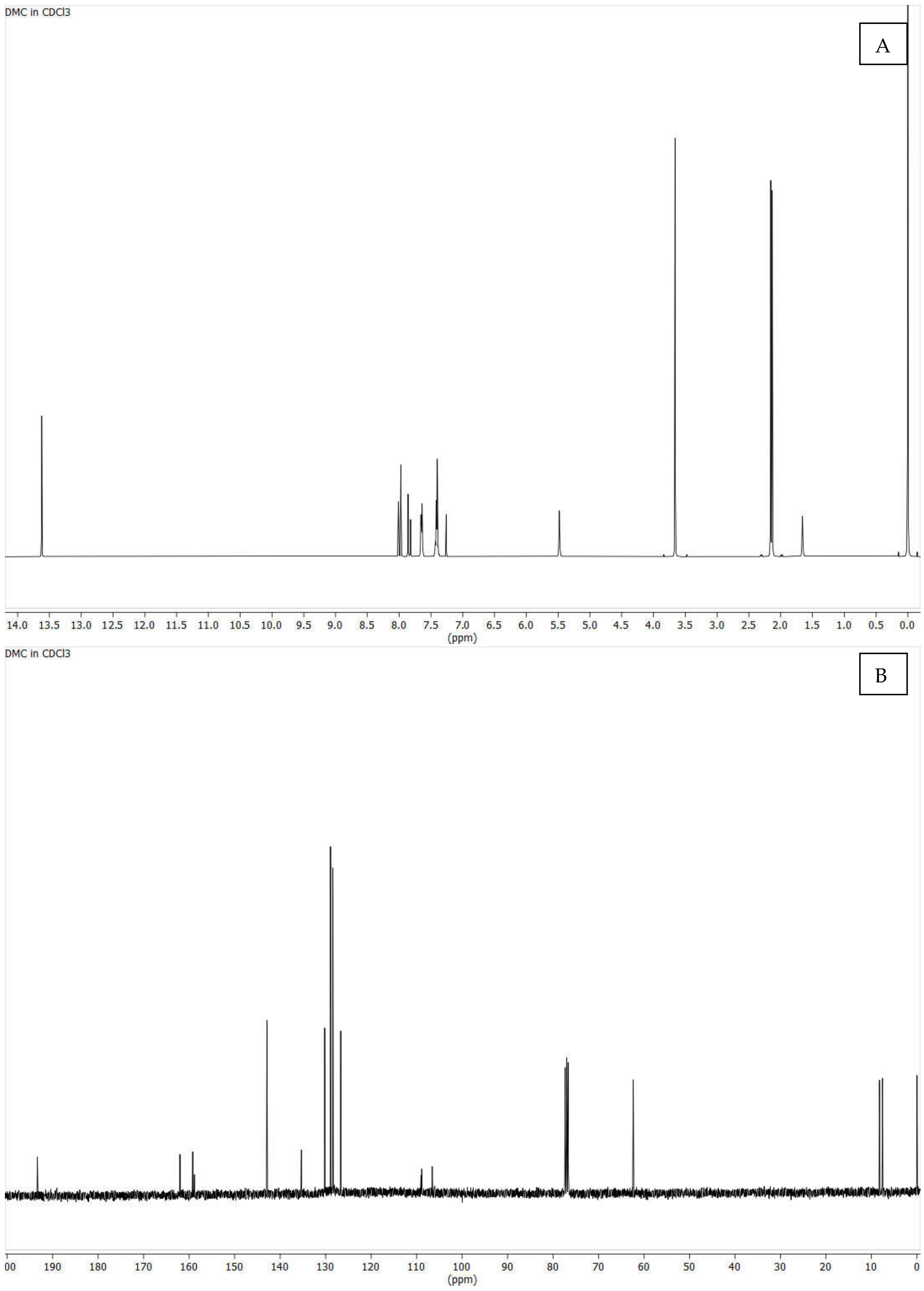

Figure S1. (A) ^{1}H-NMR and (B) ^{13}C-NMR spectrum of DMC

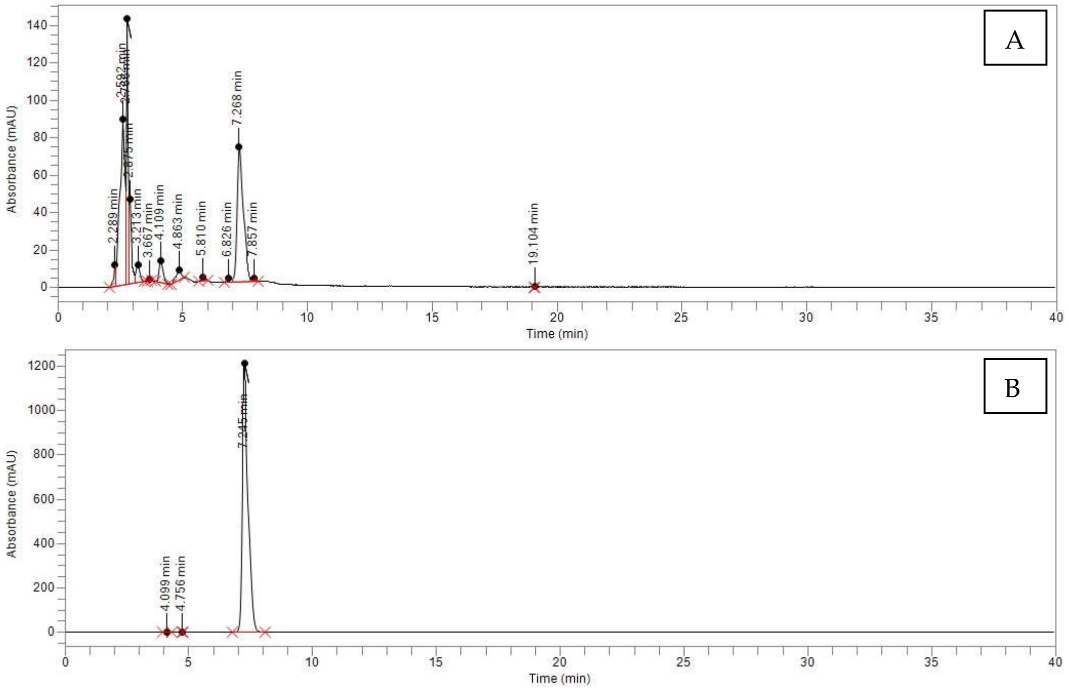

Figure S2. HPLC Chromatogram of (A) DMC-rich extract and (B) standard of DMC
